# Efficacy of self-management exercise program with spa therapy for behavioral management of knee osteoarthritis: research protocol for a quasi-randomized controlled trial (GEET one)

**DOI:** 10.1186/s12906-018-2339-x

**Published:** 2018-10-16

**Authors:** Chloe Gay, Candy Guiguet-Auclair, Bruno Pereira, Anna Goldstein, Loïc Bareyre, Nicolas Coste, Emmanuel Coudeyre

**Affiliations:** 1 Service de Médecine Physique et de Réadaptation, CHU de Clermont Ferrand, INRA, Université Clermont Auvergne, Clermont Ferrand, France; 2 Service de Santé Publique, CHU de Clermont Ferrand, PEPRADE, Université Clermont Auvergne, Clermont Ferrand, France; 3 Délégation Recherche Clinique et Innovation, CHU de Clermont Ferrand, Université Clermont Auvergne, Clermont Ferrand, France; 4 Délégation Recherche Clinique et Innovation, CHU de Clermont Ferrand, Clermont Ferrand, France; 5 Physical and Rehabilitation Medecine Department, University of Clermont Ferrand, Clermont Auvergne University, France, CHU Hôpital Nord, 61 Rue de Châteaugay – BP 30056, 63118 Clermont Ferrand, Cébazat France

**Keywords:** Knee osteoarthritis, Exercise, Self-management, Education, Spa therapy

## Abstract

**Background:**

Osteoarthritis (OA) is not limited to joint pain and stiffness, which can lead to disability; it is also linked to comorbidities such as overweight, obesity and fears and beliefs related to the pathology. The knee OA population appears more affected by these risk factors and has a lower physical activity (PA) level than the general population. The key challenge for OA treatment is increasing the PA level to decrease the risk factors.

**Methods:**

We aim to perform a prospective, multicentric, quasi-randomized controlled trial with an alternate-month design (1-month periods). People aged 50–75 years old with symptomatic knee OA (stage I-IV Kellgren and Lawrence scale) with low and moderate PA level will be included in 3 spa therapy resorts. The experimental arm will receive 5 self-management exercise sessions (1.5 h each; education, aerobics, strength training, range of motion) + an information booklet + 18 sessions (1 h each) of spa therapy treatment (STT). The active comparator arm will receive an information booklet + 18 sessions of STT. The primary outcome will be a change at 3 months in PA level (International Physical Activity Questionnaire short form score). Secondary outcomes will be function (WOMAC) pain (numerical scale), anxiety/depression (HAD), fears and beliefs about OA (KOFBeQ) and arthritis self-efficacy (ASES). The barriers to and facilitators of regular PA practice will be assessed by using specific items specifically designed for the study because of lack of any reference scale.

**Discussion:**

The study could demonstrate the impact of a self-management exercise program associated with spa therapy in the medium term by increasing PA level in people with OA. A benefit for ameliorating fears and beliefs and anxiety/depression and improving self-efficacy will also be analysed. The findings could offer new prospects while establishing best clinical practice guidelines for this population.

**Trial registration:**

ClinicalTrials.gov NCT02598804 (November 5, 2015).

**Electronic supplementary material:**

The online version of this article (10.1186/s12906-018-2339-x) contains supplementary material, which is available to authorized users.

## Background

Osteoarthritis (OA) constitutes the most frequent chronic joint disease. It contributes widely to disability and loss of autonomy in older people [1]. OA localization at the knee, hip and hand are most at risk [2]. According to the World Health Organisation (WHO), in 2020, chronic disease will be the main source of disability. This evolution is related to the increase in life expectancy due to improvements in medical technology [3].

Risk factors are multiple: heredity, overweight and trauma (sports, professional, surgery) [4, 5]. All these factors interact with each other and may contribute to worsened pain and disability and reduced mobility. With lack of any curative treatment, except prosthetic surgery, non-pharmacological treatment is essential [6].

International guidelines such as from the Osteoarthritis Research Society International (OARSI) recommend a non-pharmacological intervention associated with pharmacological treatment for pain for people with knee OA. Non-pharmacological therapies are exercise programs (strength training, aerobic activity, adjunctive range of motion, stretching and increasing physical activity (PA) level), self-management education program and weight loss if necessary [7–9]. Education and self-management exercise have a positive impact on pain, function, exercise level, weight, quality of life, and treatment adherence [10]. Self-management exercise programs have a clinical and behavioral impact [11].

People with OA have less PA level than the general population [12]. Comorbidities and risk of death are increased: history of diabetes, cancer, or cardiovascular disease and the presence of waling disability are major risk factors. People with more concerns are less active. PA practice can have two effects: ameliorating comorbidities and alleviating knee OA symptoms. However, adherence to non-pharmacological treatment is often incomplete. To be fully effective, an exercise program must be accompanied by measures to promote therapeutic adherence. Among these measures, education and information improve guideline adherence [11].

The efficacy of spa therapy in OA has been demonstrated, with good level of evidence for pain and disability, but the effect on behavioral management such as PA level is still unknown [13]. The last OARSI recommendation include spa therapy but restricted spa treatment to a subgroup of patients with generalized OA with comorbidities because of a lack of evidence [7]. Spa therapy effect is well known on pain, physical function [14, 15] and symptomatic drug consumption [16]. Short and long-term efficacy (up to 9 months) were demonstrated [16] on painful symptomatology and functional capacities in knee osteoarthritis people. Average direct costs per patient were higher in the usual treatment than mud-bath therapy in addition to usual care [17]. Mechanisms of action in spa therapy treatment are not fully understood but a combination of factors: mechanical, thermal and chemical seems to be the most evident [18]. Recent research on possible biomarkers showed a significant increase of Carboxy Terminal cross-linked Telepeptides of Type II (CTX-II), two weeks after mud-bath therapy [19]. According to Giannitti [20], mud-bath therapy decrease whole-blood level of miR-155, miR-181a, miR-146a, and miR-223 expression levels for knee osteoarthritis people. Furthermore mud-bath therapy modifies an important mediators of cartilage metabolism, the plasma levels of the adiponectin [21].

SPA therapy resorts, common in Europe, include a large sample of patients with different phenotypes from early to advanced OA stages. The spa treatment context could offer good conditions for behavioral modification and could be a special opportunity for self-management education. The spa resort is the opportunity to meet and interact with other patients and benefit from multidisciplinary medical and paramedical support for ameliorating pain and disability [22]. A combination of spa therapy, physical exercise, and self-management was found to benefit fibromyalgia symptoms [23]. Another study of low back pain, self-management exercise and spa therapy treatment (STT) is under way [24].

We aim to conduct a multicentre, prospective, quasi-randomised study to evaluate the effectiveness of a self-management exercise program associated with STT versus STT alone, on the physical activity level of patient with knee osteoarthritis, at 3 months follow up.

## Methods

### Aim

This study is aimed at people with knee OA. The main objective is to show a change in PA level 3 months after the self-management education program associated with STT. Secondary objectives are to assess the effectiveness of the self-management exercise program in terms of pain, disability, anxiety, depression, self-efficacy, fears and beliefs and barriers to and facilitators of OA and PA at 3 months, and sarcopenia after treatment (18 days).

### Study design and setting

This is a 2-year multicenter, prospective, comparative, and quasi-randomized trial. The population will be randomised according to the alternate-month design method in 2 arms: experimental and active comparator.

The design and conduct of this trial will adhere to the requirements of the Standard Protocol Items: Recommendations for Interventional Trials (SPIRIT) [25]**.** The results will be reported in accordance with the CONSORT Statement for non-pharmacologic trials [26].

### Participants and recruitment

We will recruit 142 participants, male and female, who are 50–75 years old with a diagnosis of mono or bilateral knee OA, in 3 spa therapy resorts, in France. All people already registered for STT will receive an information letter with study notification and eligibility criteria. Posters in each participating spa resort will be used for recruitment. The center is Clermont-Ferrand University Hospital associated with Royat spa center, le Mont Dore spa center and Bourbon-Lancy spa center.

Patient recruitment potential among people with knee OA in spa therapy is important. Indeed, OA represents the main disease treated by spa therapy (250,000 per year in France).

For people who meet the inclusion criteria, the research coordinator will perform the information and consent process and the physician will verify the inclusion criteria.

### Eligibility criteria

#### Inclusion criteria

- People, male or female, 50 to 75 years old
- Mono or bilateral knee OA according to American College of Rheumatology (ACR) criteria [27]
- Radiological score: stage I to IV on Kellgren and Lawrence scale
- Covered under the national health insurance
- Giving informed written consent to participate in the study

#### Exclusion criteria

- Contraindication to spa therapy
- Unstable angina
- Cardiac failure
- Behavioral disorders or comprehension difficulties making assessment impossible
- High PA level according to International Physical Activity Questionnaire (IPAQ) categorical score [28]

### Randomisation

The population was randomized according to the alternate month design method in two arms: experimental and active comparative arm. Weingarten, highlight that’s it’s possible to avoid contamination between the 2 groups with a therapeutic window between the intervention period and control period [29]. This methodology was previously used with good level of evidence in a self-management education program and low back pain treatment in a spa therapy resort [30]. Individual randomization suggests recruitment difficulties (incomplete intervention group risk) and feasibility, as well as an increase in the number of patients lost to follow-up in the control group.

**Fig. 1:**
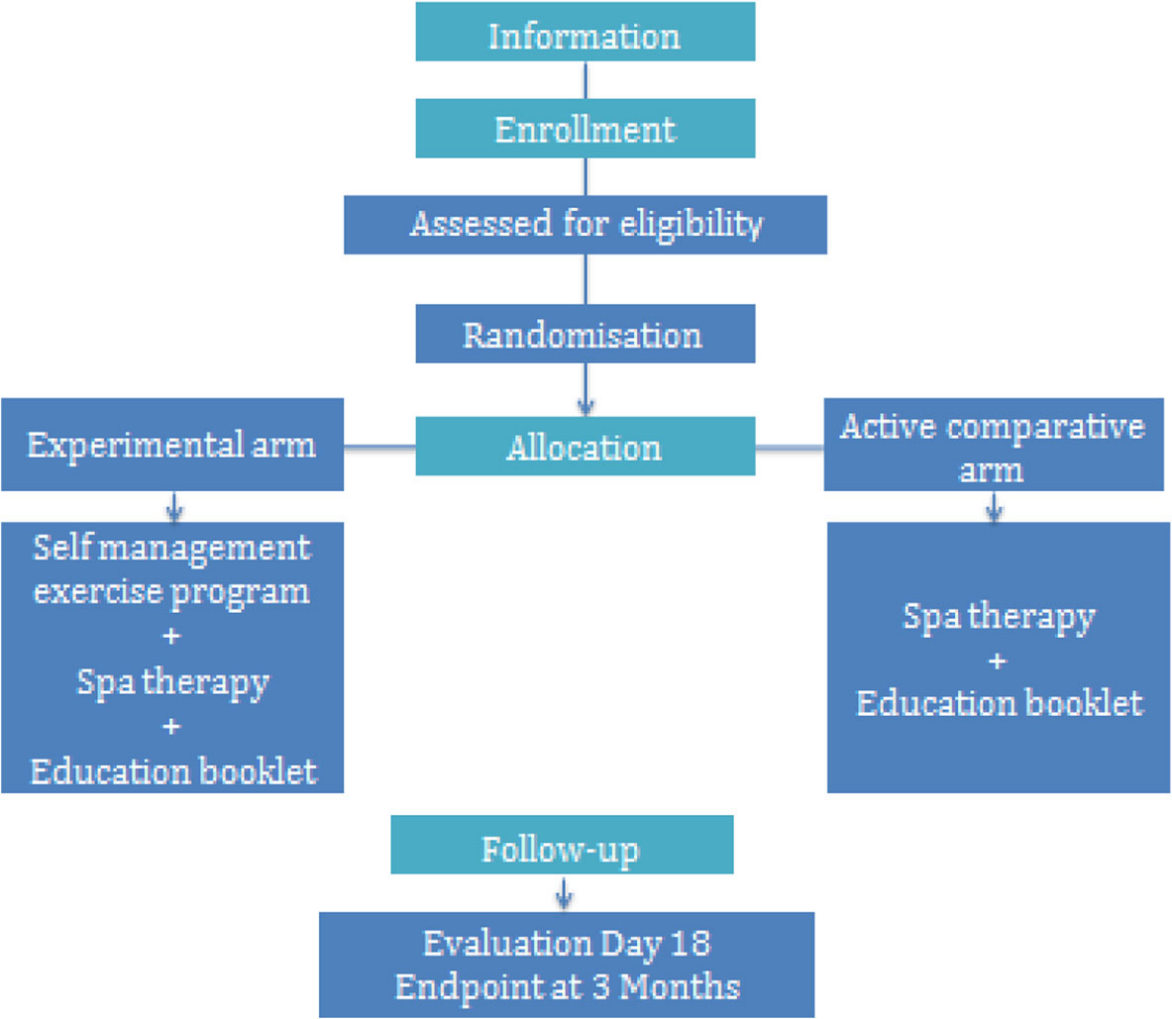
Flow of the participants through the study

**Fig. 2:**
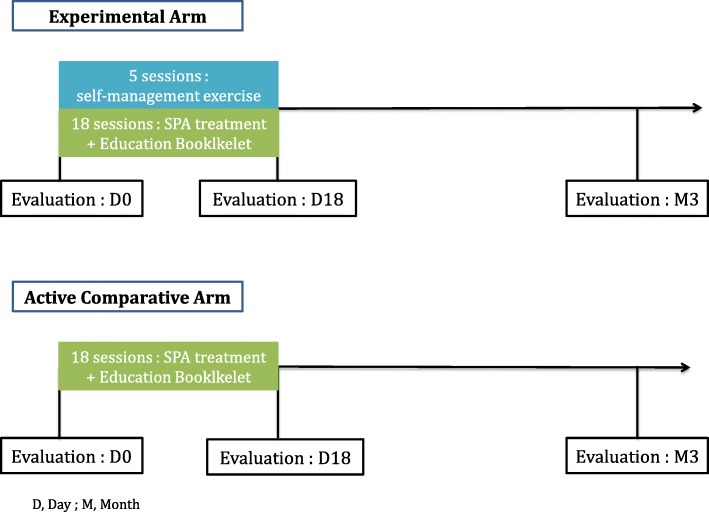
Study design

The randomisation list will be established by the methodologist in charge of the project before starting the trial. Population groups will be assigned to arms by stratified randomization by center: this randomization allows for controlling eligibility and for communicating some information relative to randomisation from the investigator and eventually other actors. A document detailing the procedures for randomisation is kept confidential. Participants will be allocated to one of two arms (Figs. 1 and 2).

### Interventions

Both groups will receive 18 sessions of STT over 3 weeks and an education booklet on the benefits of PA practice illustrated with pictures and a detailed description of the exercises to perform (Additional file 1). Only the experimental group will receive the self-management exercise program. The aim is to propose a reinforcement of the STT effect through a self-management exercise program.

The STT and the self-management exercise program will be standardised and designed by expert spa therapy physicians.

Interventions have been designed by a specialist steering committee, standardized and reproducible for the 3 centers. The steering committee consists of investigators, a spa therapist, physician and physiotherapist and the university hospital’s medical team (physician, physiotherapist, adapted PA instructor).

Meetings with all members of the steering committee are organized to obtain consensus on the therapeutic protocol describing precisely the content and the organization of the intervention taking into account the context and the expertise of the different centers involved in the study.

### Self-management exercise program

The main objective of the self-management exercise program will be to allow people to understand the importance of physical exercise practice and learn when, where and how to practice exercise, in order to adapt their PA practice according to their phenotype and integrate it long term in their daily life.

Each session will consist of 45 min of self-management education and 45 min of PA practice, with aerobic training program, strength training, range of motion and personal feedback. The exercise program will be tailored to each participant. The physiotherapist or adapted PA instructor will drive an experimental arm of 5 to 7 participants. The 5 sessions will take place on days (D) D4, D7, D10, D13 and D16.

For each workshop, a specific objective and operational objectives are defined, linked to criteria and indicators (Table 1).

**Table 1:** Self-management education program

Session	Objectives
Session 1: ᅟEvaluate the representation of the patient’s PA	- Identify the representation of PA
	- Identify the barriers to and facilitators of regular PA practice
	-Acquisition of knowledge about OA
	-Know the methods of the exercise practice
Session 2: ᅟDefining the importance of exercise in knee OA	-Name the benefits of PA in the management of knee OA
	-Identify the normal and abnormal physiological signs during the PA practice
	- Learn to adapt the exercise according to its knee OA (intensity, duration)
Session 3: ᅟPatient engagement in regular PA practice	- Determine where, when and how to exercise throughout the year
	- Include an exercise program in everyday life
	- Know the anatomical bases necessary for performing the adapted exercises
Session 4: ᅟThe consequences of stopping the exercise program	-Know the importance of maintaining exercise program on a regular and long-term basis
	-Identify factors that could facilitate or limit the continuation of the exercise program
	-Learn how to build a PA session
Session 5: ᅟKnowledge restitution	- Know the means of managing knee OA
	- Know how to live healthy with knee OA
	-Set an achievable goal

*PA* physical activity, *OA* osteoarthritis

To facilitate the workshops, different teaching tools and techniques will be used: paper board, thematic discussion, participative discussion, practical leaflets (Borg scale, deconditioning circle), information booklet, exercise demonstration, feedback, and pain coping skills.

### Education booklet

Patients will receive an information booklet containing cards with physical exercises adapted to their pathology to integrate the exercise program into their daily routine. This booklet will contain information about the pathology and PA practice with knee OA and illustrated cards with detailed descriptions of the exercises to be practiced (Additional files 1 and 2).

### SPA therapy treatment

Each spa therapy session will comprise a mineral hydrojet session at 37 °C for 15 min, the thigh manipulated under mineral water at 38 °C by a physiotherapist for 10 min, manual massages, the application of mineral-matured mud at 45 °C to the knees for 15 min and supervised general mobilisation in a collective mineral water pool at 32 °C in groups of 8 patients for 15 min of 18 sessions, for 1 h each.

The main component of mineral waters are chlorobarbonated sodium in Royat (63) and Le Mont-Dore (63) spas centers and sodium chlorides in Bourbon Lancy (71).

Both groups will be mixed with the general public in the spa center. The spa therapist will not be aware of which patients will be taking part in the clinical trial.

The experimental and active comparator arms will receive unrestricted non-pharmacological, pharmacological and usual care during the study. Medication taken during or after the intervention will be reported in the follow-up questionnaires.

### Measures

At baseline, we will collect data on sociodemographic items (age, Body Mass Index (BMI), marital status, area living, education status), anthropometric measures (Bioimpedance, Short Physical Performance Battery, Handgrip) and co-morbidities association, according to OARSI guidelines [7] (diabetes, hypertension, cardio vascular disease, renal failure; gastrointestinal (GI) bleeding, depression, or physical impairment limiting activity, including obesity).

#### Primary outcomes

The primary outcome is the proportion of participants varying to one IPAQ class at least (low-moderate or high or moderate-high) at 3 months [28]. The IPAQ short form (International Physical Activity Questionnaire) is a self-administered questionnaire which measures the physical activity level of people in a heterogeneous population as well as knee osteoarthritis people [31].

#### Secondary outcomes

● The arthritis self-efficacy influence on pain, function and other symptoms is assessed with the Arthritis Self-Efficacy Scale (ASES) [32] validation in French language is in progress NCT: 02977325 [33].

● Fears and beliefs changes are measured by the Knee OA Fears and Beliefs Questionnaire (KOFBeQ) [34].

● Physical function is assessed by the Western and McMaster Universities Osteoarthritis Index (WOMAC subscale for physical function (W-TPFS)) [35, 36].

● Pain intensity during the last 24 h and the worst pain intensity during the last month are measured by a numerical pain scale.

● Overall psychological status is assessed by the Hospital Anxiety and Depression Scale (HAD) [37, 38].

● The barriers to and facilitators of regular PA practice are assessed by 24 independent and specific items designed by a qualitative study [39], specifically for this study because of no reference scale. Each item will be coded from 0 (strongly disagree) to 4 (strongly agree). The prospective validation of this new scale is in progress.

● Use of pharmacological treatment is recorded (paracetamol, opioids, NSAIDs, intra-articular corticoid/hyaluronic acid).

### Treatment adherence

Global PA practices, specific exercises and use of the education booklet will be reported in a self-reported questionnaire at 3 months to assess level of adherence to the self-management exercise program [26]. Reasons for low adherence will be explored: therapeutic effect, novelty, social link, global health, body image, habits.

### Time-point outcomes

Study outcomes will be collected at baseline and after treatment (18 days) in spa resorts and at 3 months post-randomisation, by mailed self-reporting questionnaires. If needed, the research officer will use a telephone follow-up.

### Statistical considerations

#### Sample size estimation

According to previous works presented in literature [11], we have estimated that a sample size of *n* = 65 patients per randomized group, for a two-sided type I error at 5%, would provide 80% statistical power of detecting an absolute difference of 20% (25% vs 5.) in the primary outcome: proportion of participants varying to one IPAQ class at least (low-moderate or high or moderate-high). Finally, a total of *n* = 142 patients (71 by group) will be considered to take into account lost to follow-up (10%).

#### Statistical analysis

Statistical analyses will be conducted using SAS software (version 9.3.). A two-sided *p*-value of less than 0.05 will be considered to indicate statistical significance (except interim analysis).

Concerning the primary outcome, the comparison between groups will be analysed using Chi-squared or Fisher’s exact tests. Intention to treat (ITT) analysis will be considered for the primary outcome. Then, the analysis of the primary outcome will be completed by multivariate analysis using a generalized linear mixed model (logistic for dichotomous dependent endpoint) to take into account: (1) fixed effects covariates determined according to univariate results and to clinical relevance (for example gender, age and analgesic treatments), and (2) centre as random-effects (to measure between and within centre variability). Continuous endpoints will be compared between groups using Student’s t-test or Mann-Whitney’s test. Normality will be studied by the Shapiro-Wilk test and homoscedasticity using the Fisher-Snedecor test. As suggested by Vickers and Altman [40], the comparison between groups will be completed by linear mixed model considering randomization group and baseline values as independent parameters (fixed effects). Other categorical parameters will be analysed as described previously. Longitudinal analyses concerning repeated measures (for example ASES, KOFBeQ, WOMAC, EVA pain, fears and beliefs, assessed at baseline, after intervention and 3 months after intervention) will be studied using random-effect models (linear or generalized linear), to take into account patient as random-effect (slope and intercept), nestled in centre random-effect, while studying the fixed effects group, time and group *interaction x time*.

According to clinical relevance, sub-group analyses depending gender will be proposed after the study of sub-group x randomization group interaction in regression models (for repeated data or not). Secondarily, a per-protocol analysis will be considered. Finally, a sensitivity analysis will be performed and the nature of missing data will be studied (missing at random or not). According to this, the most appropriate approach to the imputation of missing data will be proposed (maximum bias (e.g. last observation carried forward vs. baseline observation carried forward) or estimation proposed by Verbeke and Molenberghs for repeated data). More precisely, concerning the IPAQ score, when participant will mention a number of days, hours or minutes for an activity without checking « yes » or « no » to intense, moderate or walking categories practice, a « yes » response will be imputed to this question. If participant no mentions to the practice of an activity or the number of days, hours or minutes, a « no » response will be imputed to this question.

## Discussion

In the context of an aging population, OA is a high-prevalence disease, whose prevalence will increase in the future [41]. In addition, OA is not limited to joint pain and stiffness, and the health care strategy needs to be standardized and personalized with optimized adherence to treatment [42]. This research question is important because OA lacks a cure, and few solutions to the management of knee OA are offered to patients. Treatments will be based on modifiable risk factors, such as pain, function, obesity, comorbidities, intrinsic barriers to PA practice, and sedentary time [43, 44]. The knee OA population appears to be more affected by these risk factors [45, 46], and the most severely affected patients are the less active ones [12]. The key challenge for OA treatment consists in increasing PA level to decrease the risk factors [47].

This quasi-randomized trial will be the first study to compare the effect of a self-management exercise program associated with spa therapy versus spa therapy alone. The non-invasive, adapted, tailored and original character of the intervention is a novel approach for knee OA management.

STT can improve pain and functional capacity of people with knee OA [14]. PA practice must be the key element in managing knee OA symptoms, according to recommendations [8]. However, many obstacles to exercise have been described [48], such as fears and beliefs about pain, treatment and PA [49]. Hence, changing the PA behaviour of people with knee OA is difficult [50]. Education and self-management are based on the bio-psycho-social model, effective strategies for modifying fears and beliefs and increasing adherence to treatment [51].

This protocol has some limitations. The quasi-randomized method could be a limitation, but this method is the most appropriate to avoid contamination biases between groups [29]. When assessing the effectiveness of non-pharmacological treatments such as behavioral therapy the blinding of participants and care providers is frequently impossible [52–54]. Moreover, the success of the treatment often depends on the skill and experience of care providers [26, 55, 56].

The study could demonstrate that self-management exercise programs offer a complementary therapy to the spa therapy effect could reinforce in the medium term increasing PA level and could ameliorate fears and beliefs, self-efficacy and anxiety/depression.

The findings of this trial could offer new perspectives in establishing best clinical practice guidelines for this patient population.

### Ethics and dissemination

The study protocol was approved by the medical ethics committee of South-East France (Sud-Est 6), no. 2015/CE38, and was registered at ClinicalTrials.gov (NCT 02598804 on November 5, 2015. The trial will be conducted in compliance with both Good Clinical Practices and the Declaration of Helsinki. In accordance with French law, the ethics committee of South-East France (Sud-Est 6) and the study protocol, all patients will provide written consent to participate in the study after being informed in detail about the study procedures. The written consent will be reported in the medical file. The design and conduct of this trial will adhere to the requirements of the Standard Protocol Items: Recommendations for Interventional Trials (SPIRIT). The results will be reported in accordance with the CONSORT Statement for nonpharmacological trials. The results from this study will be disseminated through peer-reviewed publications and presentations at international scientific meetings.

## Additional files

Additional file 1: Card 1 of the education booklet, English version. (JPG 230 kb)

Additional file 2: Education booklet, French version. (PDF 1196 kb)

## Abbreviations

ACR: American College of Rheumatology
ASES: arthritis self-efficacy
BMI: Body Mass Index
D: Days
HAD: Hospital Anxiety and Depression Scale
IPAQ: International Physical Activity Questionnaire short form score
ITT: Intention to treat
KOFBeQ: Knee OA Fears and Beliefs Questionnaire
M: month
OA: Osteoarthritis
OARSI: Osteoarthritis Research Society International
PA: Physical Activity
SPIRIT: Standard Protocol Items: Recommendations for Interventional Trials
STT: spa therapy treatment
WHO: World Health Organisation
WOMAC: Western Ontario and McMaster Universities Osteoarthritis Index
