# Supplementary figures and images for: Efficacy of self-management exercise program with spa therapy for behavioral management of knee osteoarthritis: research protocol for a quasi-randomized controlled trial (GEET one)
Source: BMC Complement Altern Med. 2018 Oct 16;18:279. doi: 10.1186/s12906-018-2339-x (PMC6192279; doi:10.1186/s12906-018-2339-x)

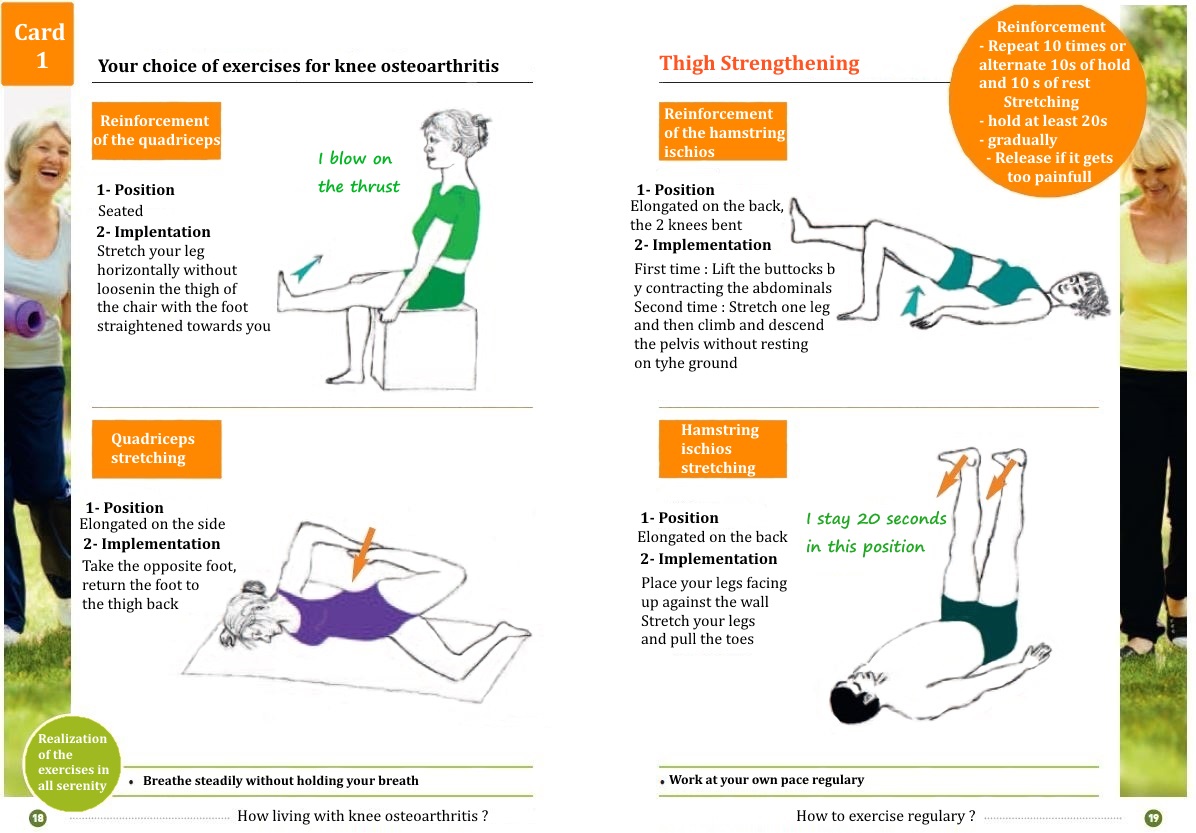

Supplement: Supplementary file 1 — Card 1 of the education booklet, English version. (JPG 230 kb) [file 12906_2018_2339_MOESM1_ESM.jpg]
