# Supplementary material for: Efficacy of self-management exercise program with spa therapy for behavioral management of knee osteoarthritis: research protocol for a quasi-randomized controlled trial (GEET one)
Source: BMC Complement Altern Med. 2018 Oct 16;18:279. doi: 10.1186/s12906-018-2339-x (PMC6192279; doi:10.1186/s12906-018-2339-x)
Supplement: Supplementary file 2 — Education booklet, French version. (PDF 1196 kb) [file 12906_2018_2339_MOESM2_ESM.pdf]

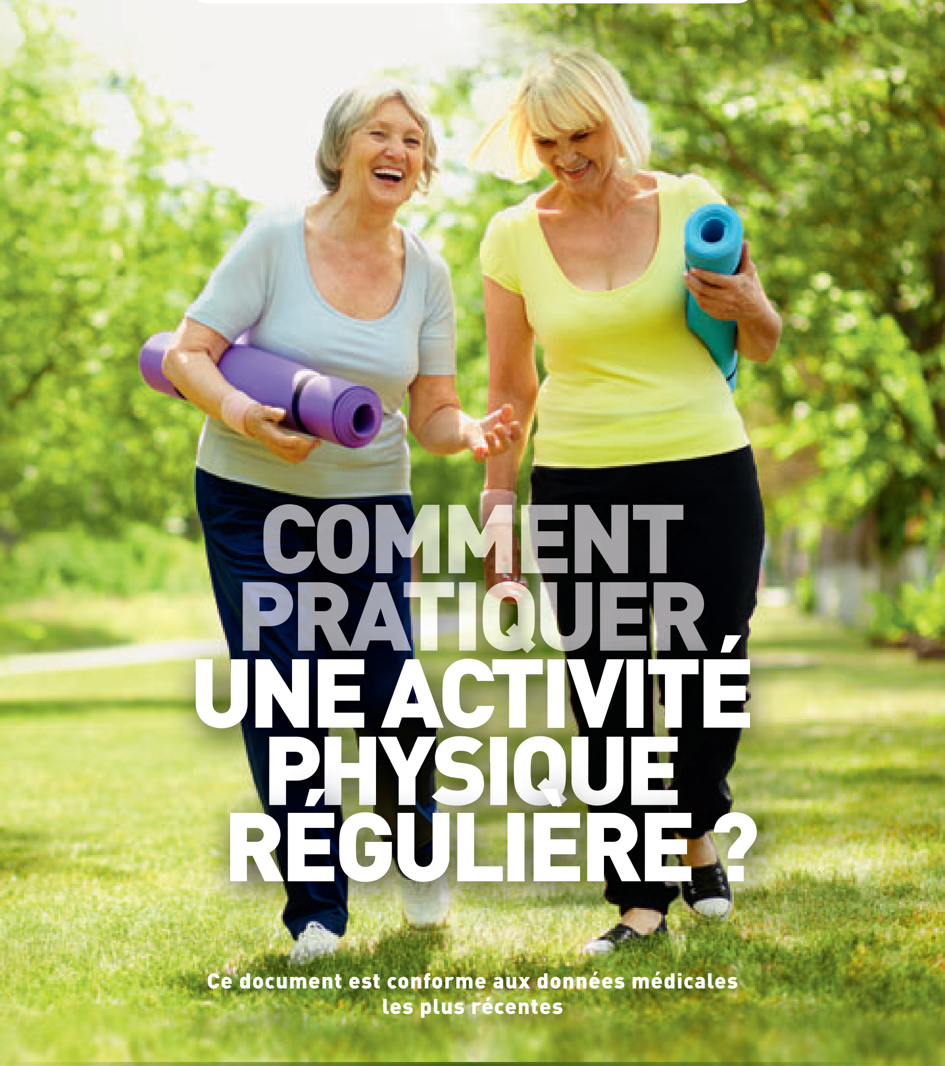

COMMENT PRATIQUER UNE ACTIVITÉ PHYSIQUE RÉGULIÈRE ?

Ce document est conforme aux données médicales les plus récentes

AVEC LE SOUTIEN FINANCIER DE

CLERMONT COMMUNAUTÉ

innovatherm – cluster auvergne thermale innovation

Cluster d'Excellence Auvergne

AUVERGNE la région juste et grande

AVEC LE SUPPORT DE

CHU CENTRE HOSPITALIER UNIVERSITAIRE CLERMONT FERRAND

Université d'Auvergne

AME2P

AFLAR

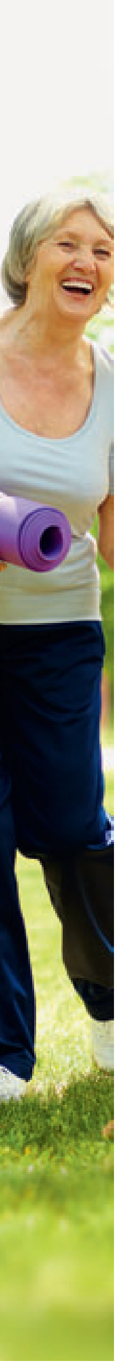

L'éditeur décline toute responsabilité concernant les informations contenues dans ce document.

Les conseils donnés par les auteurs sont à titre purement informatifs et ne doivent pas être utilisés sans avis médical.

Première édition 2015

Ce document a été établi par Chloé GAY sous la direction du Pr Emmanuel Coudeyre.

L'ensemble des informations contenues dans ce document sont issues d'études scientifiques et a été validé par plusieurs sociétés savantes.

Remerciements aux soignants et patients qui ont participé à la relecture de ce guide

Introduction

Vous souffrez de gonarthrose, ce document vous concerne. Il vous est destiné !
Il a été conçu par une équipe médicale pluridisciplinaire (Médecin de Médecine Physique et de Réadaptation, Médecin Généraliste, Rhumatologue) et paramédicaux (Masseur- kinésithérapeute, Professeur d'activité physique adaptée, Ergothérapeute, Infirmière, Psychologue).

Vos attentes

Un certain nombre d'études scientifiques ont été réalisées afin de mieux comprendre les attentes du patient gonarthrosique. Il en ressort principalement trois types d'attentes : une amelioration de la douleur, une amélioration des capacités fonctionnelles conduisant à une amélioration de la qualité de vie des patients et un retour au mode de vie antérieur.

Appropriez-vous ce livret et devenez l'acteur principal de la prise en charge de votre arthrose! L'activité physique agit sur vos douleurs et vos raideurs articulaires!

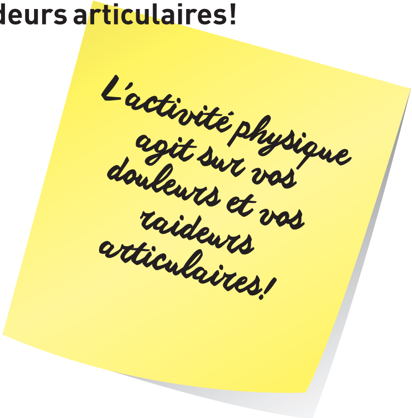

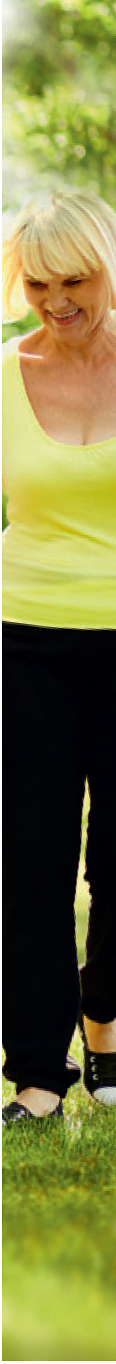

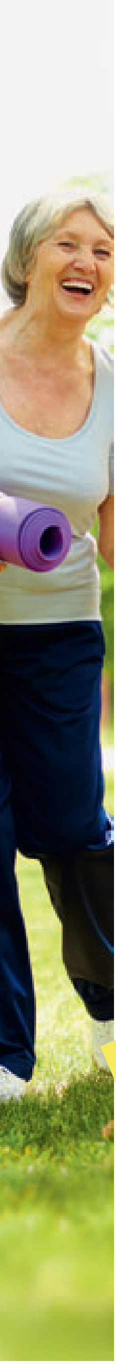

L’arthrose c’est quoi ?

L’arthrose est une dégénérescence du cartilage présent au sein de vos articulations. Le cartilage est un tissu vivant qui recouvre les surfaces osseuses de toutes vos articulations. Il a principalement deux rôles :

- assurer le glissement et donc la mobilité des articulations
- amortir les chocs entre les os de l’articulation

Lorsqu’il n’y a plus suffisamment de cartilage, les surfaces osseuses entrent en contact, ce qui est à l’origine de vos douleurs.

L’arthrose est une pathologie chronique mais bénigne qui évolue, dans la grande majorité des cas, lentement (sur plusieurs années) et par poussées.

Toutes les articulations peuvent être atteintes mais certaines le sont plus fréquemment comme le genou et la hanche.

L’objectif du traitement est d’améliorer votre qualité de vie.

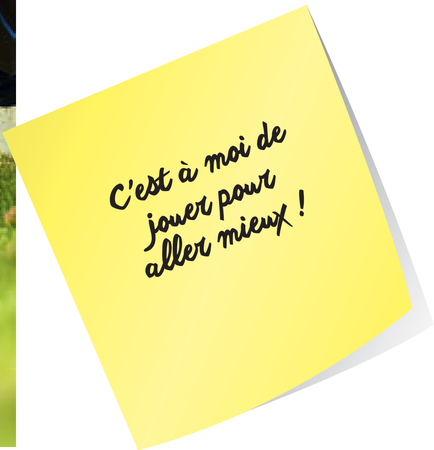

Contexte

L'arthrose touche plus souvent les femmes que les hommes, ainsi que les personnes ayant une surcharge pondérale ou des antécédents traumatiques.

Démarche diagnostique

Le diagnostic est retenu grâce aux symptômes que vous décrivez associés à l'examen clinique (douleur, gonflement, déformation du genou). L'analyse de vos radiographies permet de confirmer le diagnostic.

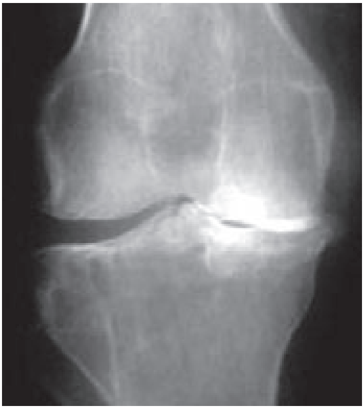

Arthrose évoluée du genou

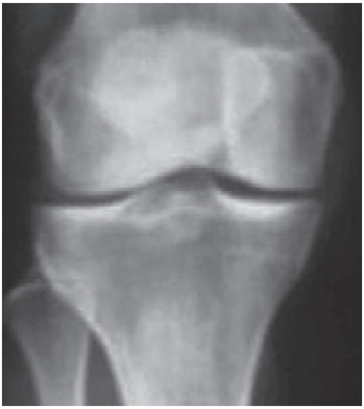

Genou normal

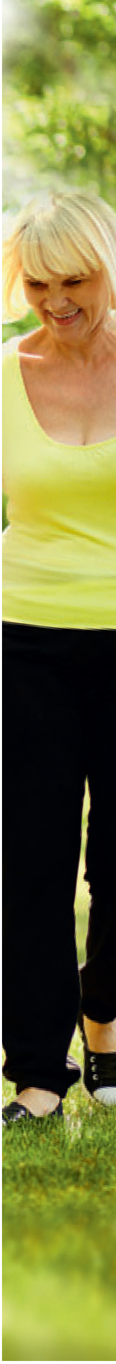

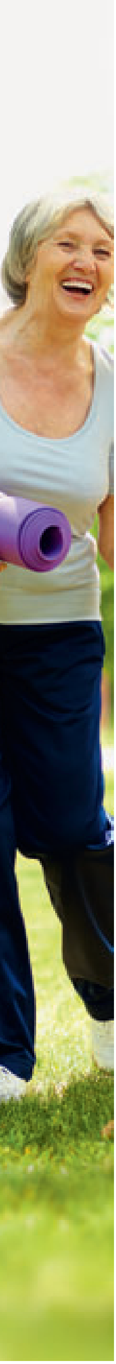

Votre genou

Le genou est une articulation supportant l'ensemble du poids du corps ; il est constitué de trois surfaces osseuses :
- l'extrémité inférieure du fémur (os de la cuisse)
- l'extrémité supérieure du tibia (un des deux os de la jambe)
- la face articulaire de la rotule

Ces surfaces osseuses, dites articulaires, sont recouvertes de cartilage. De plus, entre le fémur et le tibia s'interposent les ménisques interne et externe. L'articulation est renforcée par les ligaments latéraux et les ligaments croisés.

Le genou permet essentiellement des mouvements de flexion-extension. Cette mobilité est assurée par deux principaux groupes musculaires :
- le quadriceps, en avant, qui permet de tendre la jambe
- les ischio-jambiers, en arrière, qui permettent de plier la jambe.

Ces deux groupes musculaires jouent également un rôle majeur dans la stabilité et le contrôle de votre genou. C'est pour cette raison que l'un des objectifs de la rééducation est le renforcement musculaire.

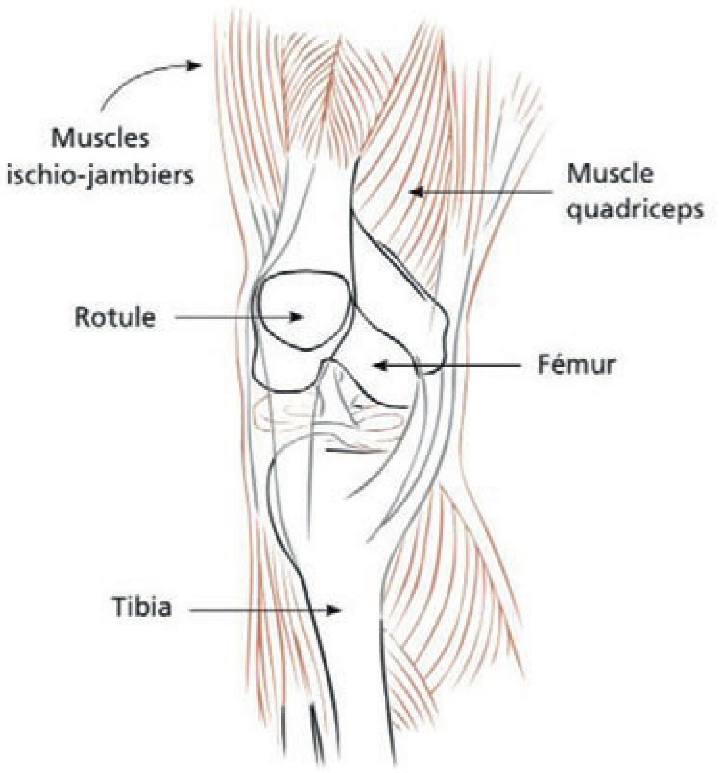

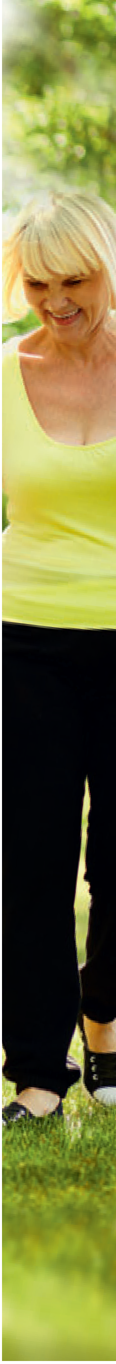

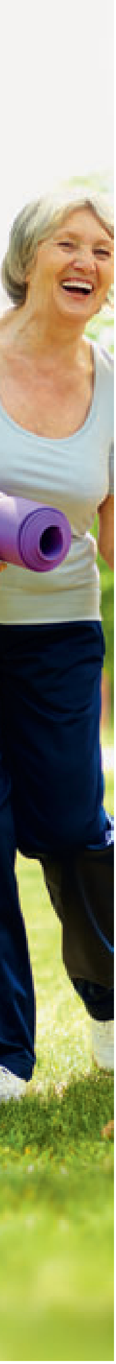

Comment traiter votre arthrose

La prise en charge de l'arthrose doit associer différents traitements médicamenteux et non médicamenteux. Ces traitements ont fait l'objet de nombreuses études scientifiques et sont validés par la pratique, permettant ainsi de proposer les recommandations suivantes. La prise en charge associe un traitement de fond et un traitement des poussées.

Traitement de fond

Il vise à stabiliser la progression de la dégénérescence du cartilage et par conséquent à retarder une éventuelle intervention chirurgicale.

Mesures non médicamenteuses

- **1) La pratique d'une activité physique régulière** est capitale. Ses bienfaits sont largement démontrés pour l'arthrose comme pour de nombreuses maladies chroniques.
- **2) La perte de poids** est essentielle. Elle a pour objectif d'obtenir un Indice de Masse Corporelle (IMC = Poids (kg)/Taille(m)^2) inférieur à 28. Cela permet de diminuer la charge supportée par votre genou à chaque pas que vous effectuez. Pour cela vous pouvez vous faire aider par une diététicienne.

Pratiquer une activité physique améliore votre condition physique

du genou ?

Je me prends en main !

- **3)** Le port d'une **orthèse de genou** (genouillère) ou d'une orthèse plantaire (semelle) peut vous être préconisé afin d'améliorer la stabilité de votre genou et de décharger les zones douloureuses. La pose de bandes élastiques adhésives (taping) peut également être proposé, les objectifs étant similaires à celui d'une orthèse.
- **4) L'utilisation de cannes** (canne simple, canne béquille ou anglaise) a un intérêt pour limiter les contraintes sur le genou, en particulier au cours des périodes douloureuses. Vous devez tenir la canne du côté le moins douloureux.

La pratique d'une activité physique régulière doit associer :

- **Des exercices spécifiques** et adaptés à chacun en fonction du type de déformations et de douleurs que vous présentez. Ils vous seront enseignés par un professeur d'activité physique adaptée ou par un masseur kinésithérapeute qui vous montrera des exercices qu'il faudra absolument poursuivre à domicile pour augmenter leur efficacité
- **Des exercices d'endurance** qui peuvent être réalisés soit en charge comme la marche, soit en décharge tels que le vélo d'appartement, l'aquagym ou la rééducation en piscine si les douleurs sont plus intenses.

Il est bien démontré que ces programmes d'exercices ont une efficacité comparable. Associer des exercices spécifiques à un travail d'endurance augmentent leur efficacité.

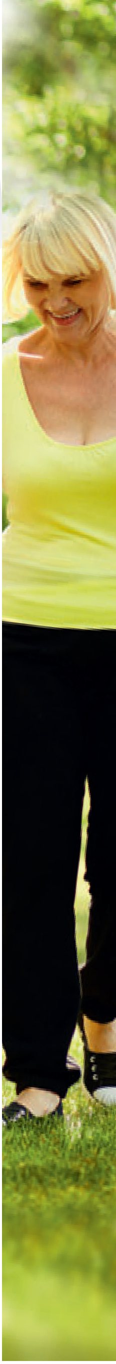

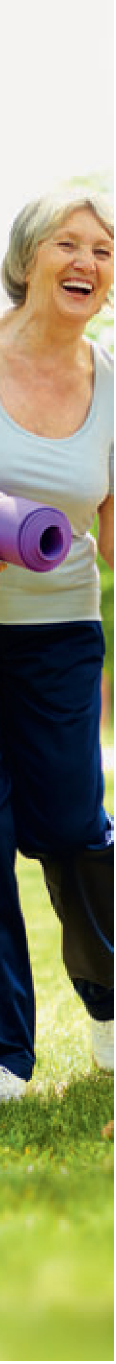

Comment traiter votre arthrose

Mesures médicamenteuses

On peut avoir recours à des traitements ayant pour objectif de protéger le cartilage, soit sous la forme de comprimés (traitement anti arthrosique symptomatique d'action lente), soit sous la forme d'injection dans l'articulation (acide hyaluronique) : on parle alors de visco-supplémentation.

Traitement des poussées

Mesures non médicamenteuses

Le but est de limiter les contraintes sur votre genou en béquillant à l'aide de canne(s) simple(s) ou anglaises, sans mettre votre articulation au repos complet, ni l'immobiliser. Vous pouvez également appliquer des packs de froid dans un linge régulièrement sur votre genou afin de diminuer la douleur de l'oedème. Vous pouvez par exemple utiliser un sac de petits pois congelés entouré d'un linge.

Mesures médicamenteuses

- **Médicaments contre la douleur** (dits « antalgiques »), ces traitements aident à contrôler la douleur, leur puissance sera adaptée à l'intensité de votre douleur ; ils doivent être pris régulièrement selon votre ordonnance et de façon générale régulièrement toutes les 4 à 6 heures.

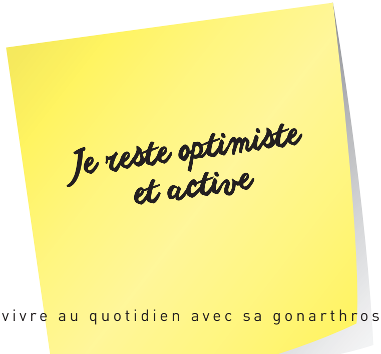

du genou ?

- **Anti-inflammatoires,** ils sont utilisés en deuxième intension, le moins longtemps possible en cure courte et la plus petite dose possible. A appliquer soit en pommade localement, soit en comprimé(s) oralement.
- Enfin, en cas « d'épanchement » dans l'articulation, on peut avoir recours à **une ponction avec injection d'anti-inflammatoire** à base de cortisone.

Pour un certain nombre de personnes, ces divers traitements peuvent être insuffisants (variable selon les individus et leur mode de vie). Le temps est peut être alors venu d'envisager la prothèse.

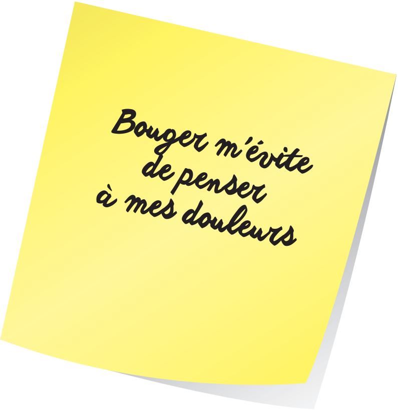

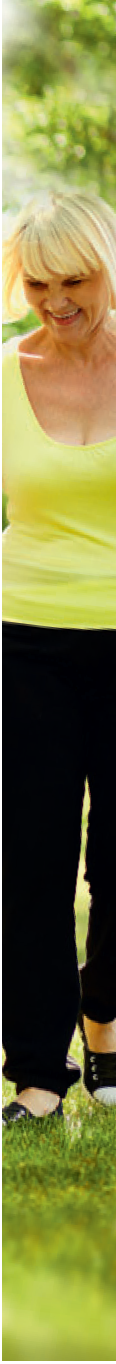

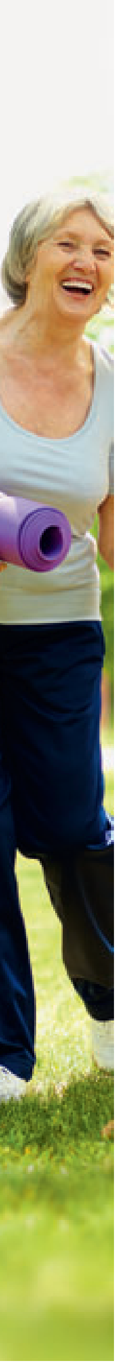

Comment pratiquer des exercices

Pourquoi faire de l'exercice ?

Tout comme votre traitement médicamenteux, l'exercice et l'activité physique ont un rôle primordial dans le traitement de votre gonarthrose.
Les douleurs causées par l'arthrose vous on fait diminuer votre activité physique et/ou vos activités quotidiennes. Les conséquences sont une diminution de votre condition physique (diminution de la force musculaire, de la tolérance à l'effort et augmentation de la raideur).
La reprise d'une activité physique et la pratique d'exercices plus adaptés vous aideront à :

- diminuer vos douleurs
- avoir une meilleure stabilité de vos articulations en augmentant ou maintenant votre force musculaire
- avoir une meilleure amplitude de vos mouvements en augmentant votre souplesse et votre mobilité
- gérer votre poids si besoin
- améliorer votre qualité de vie

Vous serez à nouveau capable de faire plus de choses, de reprendre confiance et ainsi diminuer la gene fonctionnelle occasionnée par l'arthrose. L'activité physique régulière contribue à la reduction des risques cardiovasculaires, de l'ostéoporose, de l'obésité, du diabète de type II et de certains cancers.

J'adapte ma pratique en fonction de mes symptômes

La régularité sera bien plus payante que l'inactivité !

adaptés à votre gonarthrose

Quelles sont les activités que vous pouvez faire ?

La pratique doit être progressive et adaptée à votre situation
Pour votre arthrose du genou, il est conseillé de pratiquer :

- des exercices de renforcement musculaire : **Fiches ressources N° 1, 2, 3**
- des étirements : **Fiches ressources N° 1, 2, 3**
- des exercices d'équilibre: **Fiche ressources N° 4**
- des exercices de mobilité articulaire : **Fiche ressources N° 4**
- des exercices d'endurance : **Fiche ressources N° 5**

La pratique d'exercice d'endurance

Avoir une bonne endurance vous permettra d'être plus à l'aise pour réaliser vos activités quotidiennes. Vous serez moins fatigué et moins essoufflé. **Exemples d'activités** : marche, vélo, natation **Fiche ressources N° 5**

Voici quelques conseils de pratique :

- Il est recommandé de faire 30 min par jour d'activité d'endurance que vous pouvez moduler à votre guise (par exemple, 1 fois 30 min ou 3 périodes de 10 min)
- L'intensité est modérée, vous êtes capable de répondre à quelqu'un mais pas d'entretenir une discussion
- D'une durée suffisante 30' à 45' : pour une bonne rentabilité énergétique (consommation des graisses par le muscle)
- Augmentez progressivement la durée et l'intensité de pratique en fonction en fonction de votre forme
- Pensez à boire de l'eau régulièrement

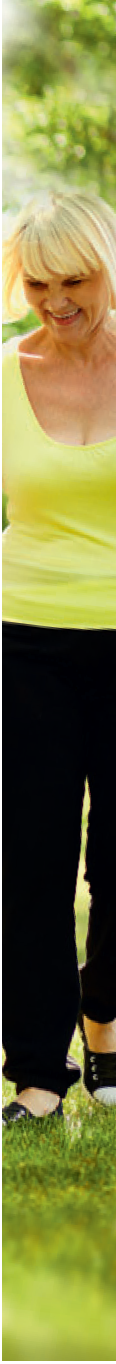

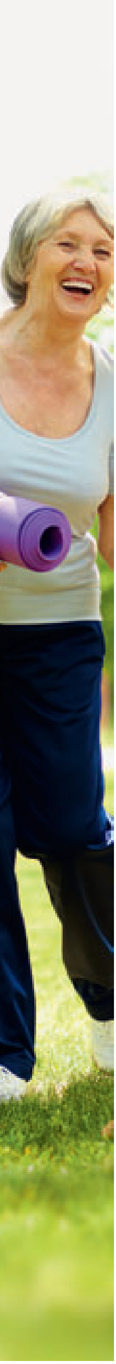

Comment pratiquer des exercices

Echelle de perception de l'effort **(Adaptation de l'échelle de Borg)**
Intensité de la sensation (essoufflement et fatigue)

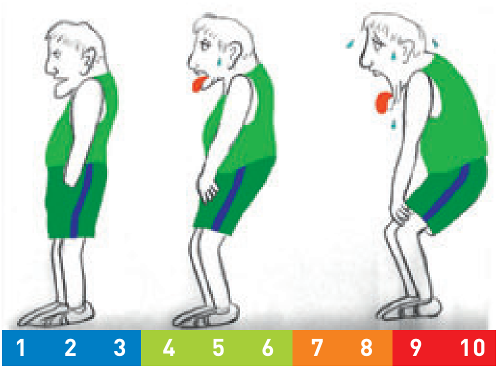

0 - rien du tout
1 - Très légèrement
2 - Légère
3 - Moédére
4 - Un peu sévère
5 - Sévère
6 - Plus sévère
7 - Très sévère
8 - Très sévère
9 - Très très sévère
10 - Maximale

Il est recommandé de pratiquer vos exercices à une intensité de sensation comprise entre **4 à 6 sur 10**

Comment se servir de l'échelle de perception de l'effort ?

- Débutez à un rythme qui vous semble confortable
- Arrivez à un essoufflement léger à modéré

Il est normal de **transpirer** et de sentir votre **corps avoir chaud** et d'avoir de **légères douleurs musculaires ou articulaires**.
→ Si vous n'avez aucun de ces symptômes, il se peut que votre intensité d'effort soit insuffisante.

Consignes de sécurité : Si vous sentez des douleurs à la poitrine, des douleurs intenses dans les articulations, de l'étourdissement, des vertiges ou des palpitations, STOPPEZ l'activité et demander l'avis d'un professionnel de santé.

adaptés à votre gonarthrose

La pratique d'exercice de renforcement musculaire

Ces exercices vont vous permettre de préserver et d'améliorer votre force musculaire. Vous avez besoin d'une bonne condition musculaire pour réaliser vos activités quotidiennes et rester autonome. Votre articulation du genou est maintenue par les ligaments et les muscles. En renforçant les muscles autour de l'articulation, vous augmentez la stabilité de votre genou et ainsi diminuer les douleurs liées à l'arthrose. Des exercices spécifiques à l'arthrose du genou vous sont proposées, c'est une partie essentielle pour un programme complet :

- **Fiches ressources N° 1, 2, 3**

Voici quelques conseils pour réaliser ces exercices :

- Les réaliser lentement (3 à 5 secondes par mouvement)
- Faire 10 répétitions, les 8 premières sont faciles, les 2 dernières doivent être plus difficiles
- Lorsque vous faites les 10 répétitions facilement, ajouter une série afin d'atteindre la bonne charge de travail
- Au cours des exercices, pensez à bien respirer et à souffler sur la poussée du mouvement

Il est possible de réaliser ces exercices tous les jours, nous vous conseillons de les faire au moins trois fois par semaine.

Je tonifie et renforce mon genou !

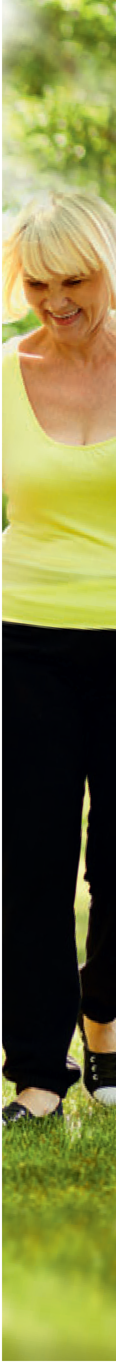

Comment faire en pratique ?

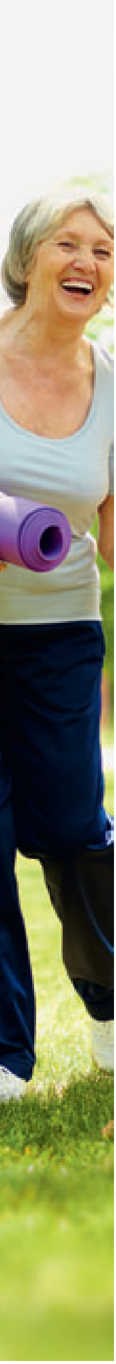

A quel moment je pratique mon exercice ?

- Réservez une période de la journée qui convient bien et donnez priorité à l'exercice. (par exemple avant la douche)
- Attendez au moins une heure après les repas

A quel endroit je pratique ?

- Choisissez un endroit agréable
- N'hésitez pas à adapter le programme en fonction des saisons, si vous aimez les activités extérieures, planifier une activité pour chaque saison
- Songez aussi à des activités que vous pouvez faire en intérieur car elles sont indépendantes des conditions météorologiques
- Renseignez vous sur les programmes d'exercice proposés près de chez vous (municipalité, association, salle de sport)
- Demandez à vos voisins et amis quels types d'exercice ils pratiquent et proposez leur de vous joindre à eux

Comment je pratique ?

- Choisissez un mode d'exercice que vous aimez
- Privilégiez une tenue pour la pratique sportive (confort chaussures et vêtements, tenue adapté à la température)
- N'hésitez pas à vous faire prescrire des semelles ou une genouillère si nécessaire
- Pensez à utiliser l'échelle de perception de l'effort ainsi que les fiches ressources
- Pensez à vous hydrater régulièrement
- Privilégier la régularité à l'intensité
- L'activité de doit pas devenir une contrainte mais rester un PLAISIR

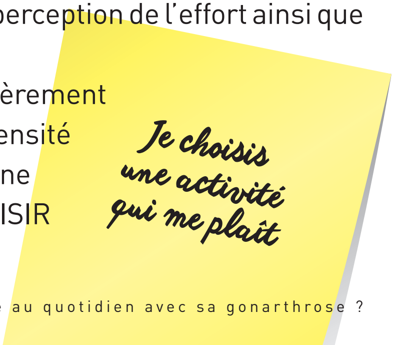

Quelle est l'importance de maintenir sa pratique sur le long terme ?

Pour conserver les effets des efforts que vous avez produits, il faut persévérer. En effet, après seulement quelques jours d'inactivité, vous commencez à perdre un peu de force et d'endurance musculaire.

C'est pourquoi, si vous rencontrez des problèmes qui pourraient représenter une barrière au maintien de votre programme d'exercice, parlez en à un de vos professionnels de santé qui vous aidera à trouver des solutions.

La pratique d'activité physique régulière est essentielle mais non suffisante à elle seule. Les effets thérapeutiques seront majorés si vous l'associez aux autres traitements recommandés dans l'arthrose :

- Une perte de poids si vous êtes en surpoids
- Le port de semelles et orthèses (genouillères)
- Un traitement médicamenteux

Vos taches de vie quotidiennes seront facilitées !

J'entretiens ma mobilité articulaire

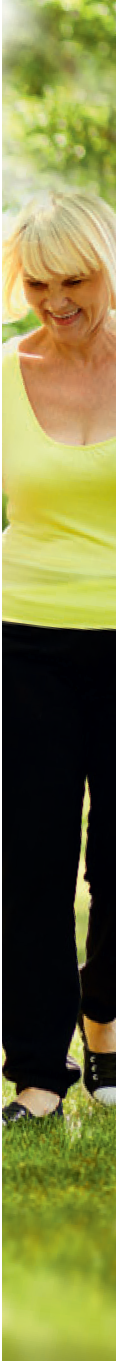

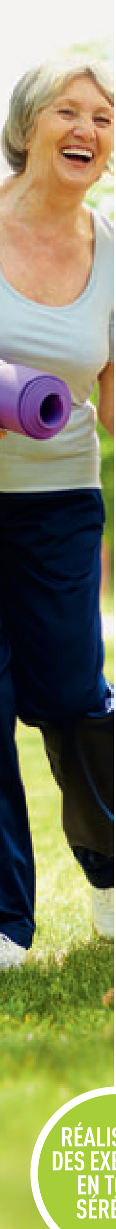

Votre choix d'exercices pour l'arthrose du genou

RENFORCEMENT DES QUADRICEPS (AVANT)

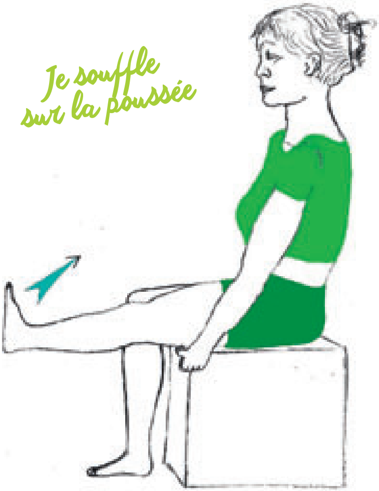

1-Position
Assise

2-Réalisation
Tendez la jambe à l'horizontale sans décoller la cuisse de la chaise avec le pied redressé vers vous

ETIREMENT DES QUADRICEPS

1-Position
Allongé sur le coté

2-Réalisation
Prenez le pied opposé, ramenez le pied vers la fesse et tirer la cuisse en arrière

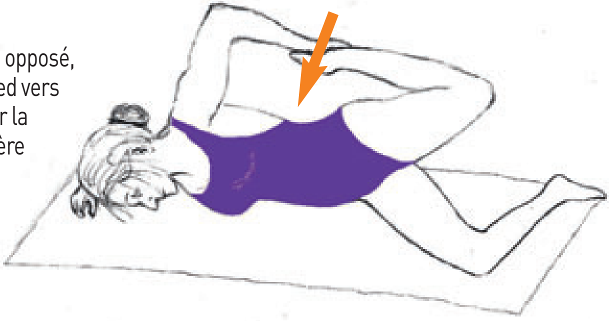

RÉALISATION DES EXERCICES EN TOUTE SÉRÉNITÉ

• Respirez de façon régulière ne pas retenir son souffle).

Renforcement **cuisses**

RENFORCEMENT
Répétez **10 fois**
OU alternez **10 s de maintien et 10 s de repos**

ETIREMENTS
- Maintenez **au moins 20 s**
- Etirez progressivement
- Relâchez si ça devient trop douloureux

RENFORCEMENT DE ISCHIO-JAMBIERS (ARRIÈRE)

1-Position

Allongé sur le dos, les 2 genoux pliés

2-Réalisation

1er temps = Soulever les fesses en contractant les abdominaux

2nd temps = tendre une jambe puis monter et descendre le bassin sans reposer au sol

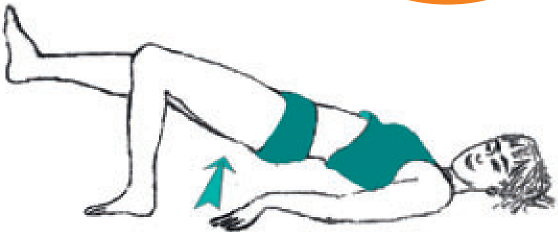

ETIREMENT DES ISCHIO-JAMBIERS

1-Position

Allongé sur le dos

2-Réalisation

Placez-vous au sol les jambes dirigées vers le haut contre le mur

Tendez les jambes et tirez les pointes de pieds vers vous

Je reste 20 secondes dans cette position

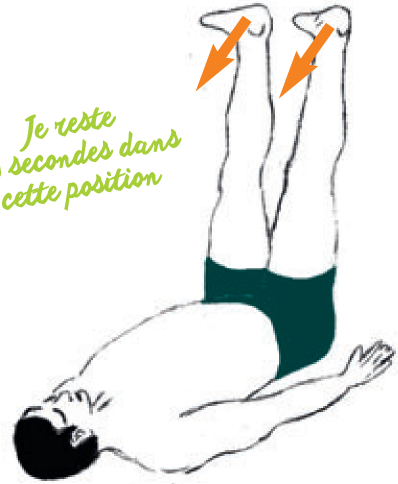

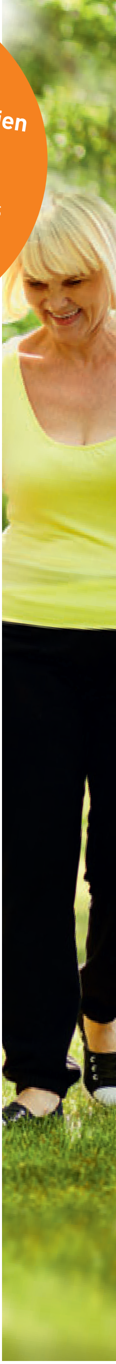

- Travaillez à votre rythme et régulièrement.

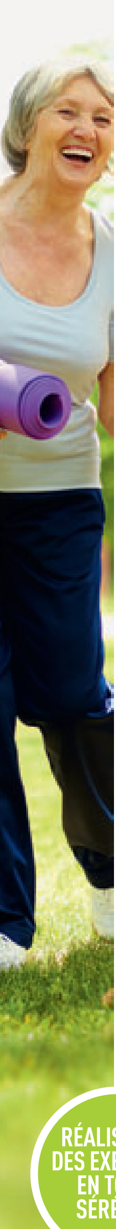

Votre choix d'exercices pour l'arthrose du genou

RENFORCEMENT DES MOLLETS

1-Position
Debout, face au mur

2-Réalisation
Montez sur demi-pointes avec les 2 pieds

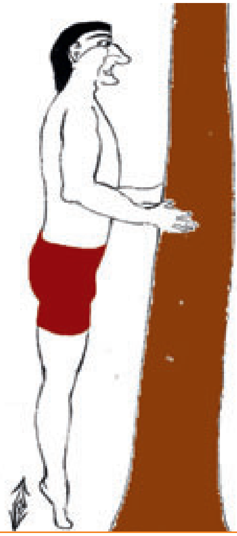

ETIREMENT DES MOLLETS

1-Position
En fente, les mains contre le mur

2-Réalisation
Pliez le genou vers l'avant et descendez le bassin vers le bas de façon à ressentir l'étirement le long du mollet

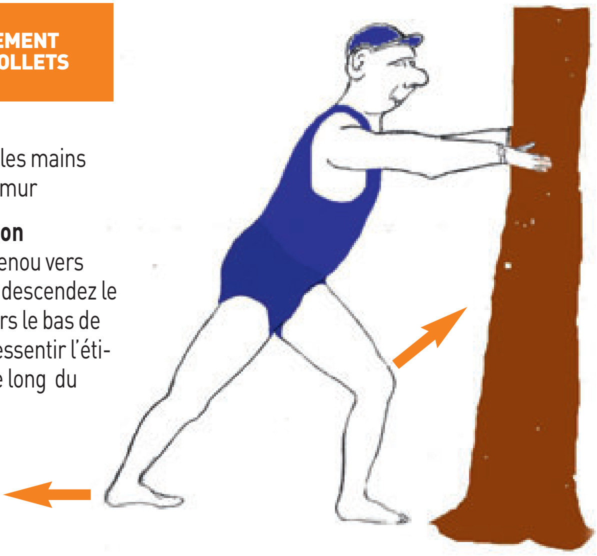

RÉALISATION DES EXERCICES EN TOUTE SÉRÉNITÉ

- Augmentez progressivement le nombre de séries en ajoutant une série de 10 quand vous parvenez à les réaliser sans difficulté.

Renforcement **mollets**

RENFORCEMENT
Répétez **10 fois**
OU alternez **10 s de maintien et 10 s de repos**

ETIREMENTS
- Maintenez **au moins 20 s**
- Etirez progressivement
- Relâchez si ça devient trop douloureux

RENFORCEMENT DU MUSCLE AVANT DU MOLLET & DE LA CUISSE

1-Position
Assise

2-Réalisation
Ecrasez le talon au sol

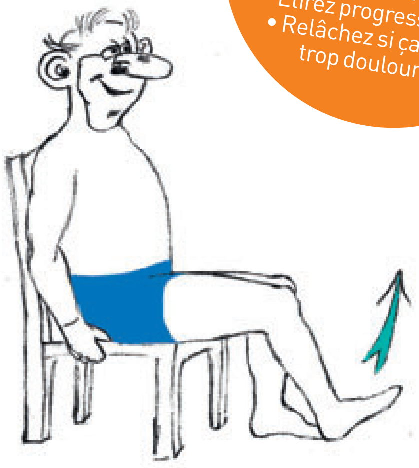

EXTENSION DU GENOU

1-Position
Allongé sur le dos, une serviette roulée sous le genou

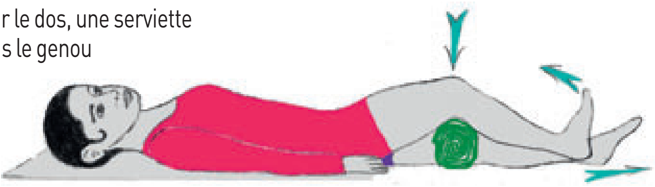

2-Réalisation
Contractez les muscles de telle sorte à écraser la serviette avec le genou

Poussez le talon et ramenez la pointe de pied vers soi sans décoller le talon

- Les étirements des muscles de la chaine postérieure de la jambe permettent de lutter contre la flexion durable du genou due à la douleur.

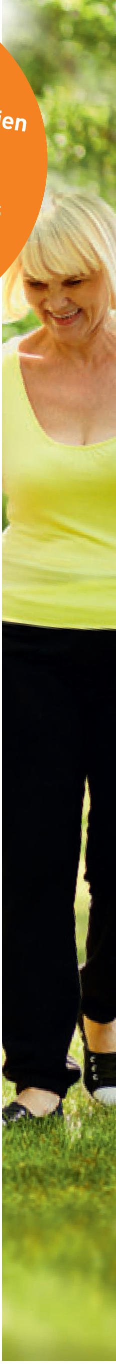

Votre choix d'exercices pour l'arthrose du genou

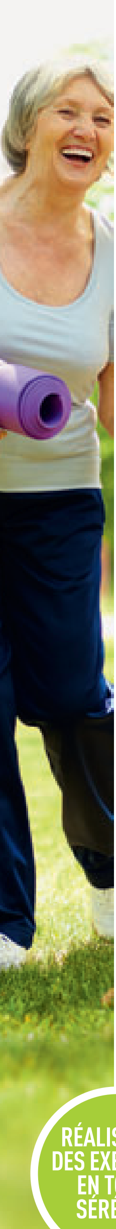

RENFORCEMENT DES ABDUCTEURS

1-Position
Accoudé, reposant la tête sur la main opposée

2-Réalisation
Stabilisez-vous en fléchissant un peu la jambe qui repose sur le sol et en vous aidant de la main

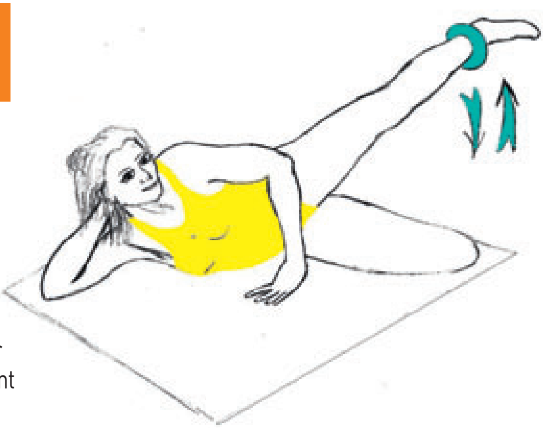

ETIREMENTS DES ABDUCTEURS

1-Position
Assis, une jambe fléchie par dessus

2-Réalisation
Avec vos mains, attrapez le genou et rapprochez- le de votre poitrine

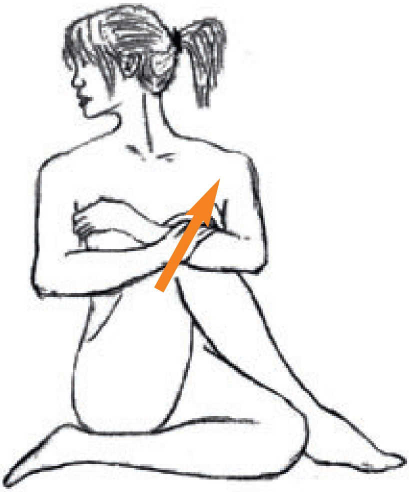

RÉALISATION DES EXERCICES EN TOUTE SÉRÉNITÉ

• Renforcez les muscles abducteurs et adducteurs (intérieurs et extérieurs du genou) permet d'améliorer la stabilité du genou.

Renforcement **cuisses**

RENFORCEMENT
Répétez **10 fois**
OU alternez **10 s de maintien et 10 s de repos**

ETIREMENTS
- Maintenez **au moins 20 s**
- Etirez progressivement
- Relâchez si ça devient trop douloureux

RENFORCEMENTS DES QUADRICEPS

1-Position
Assise

2-Réalisation
Placez la cheville au-dessus de l'autre

Monter la jambe de dessous et s'y opposer avec celle placée au dessus

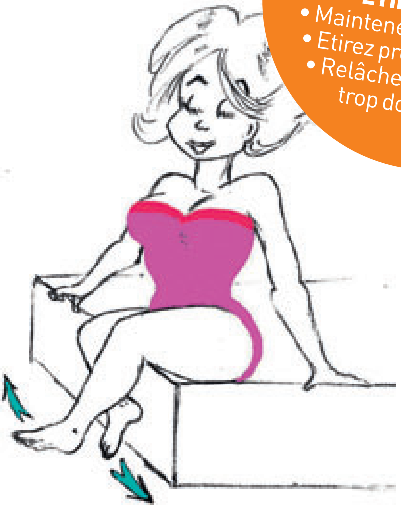

ETIREMENTS DES ABDUCTEURS

1-Position
Debout, jambes écartées

2-Réalisation
Les jambes écartées, penchez-vous sur un côté en fléchissant la jambe

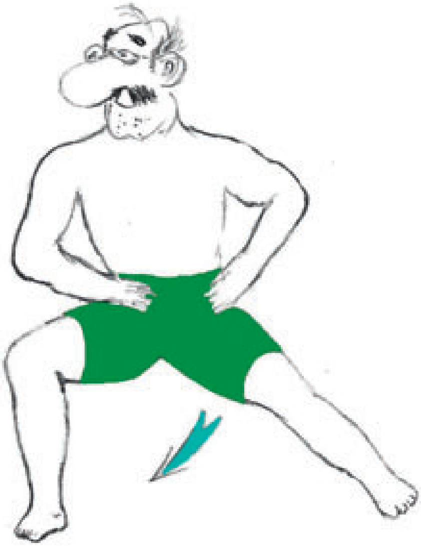

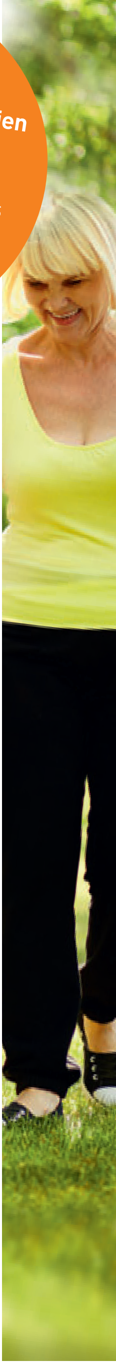

- Une meilleure stabilité de l'articulation permet un meilleur équilibre et une protection efficace du genou.

FICHE 4

Votre choix d'exercices pour l'arthrose du genou

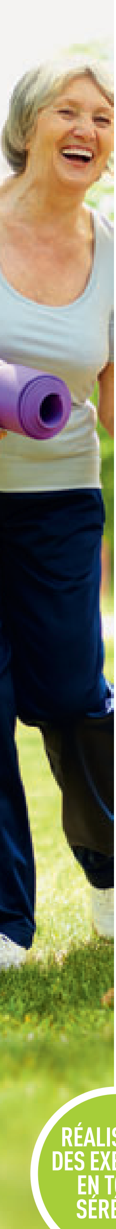

SOUPLESSE DU GENOU

1-Position
Assis, jambes tendues

2-Réalisation
A l'aide d'un tissu que vous placez derrières les orteils, tirez vers vous en gardant le dos droit

ASTUCE
Faites une jambe près l'autre. Maintenez l'étirement 20 secondes

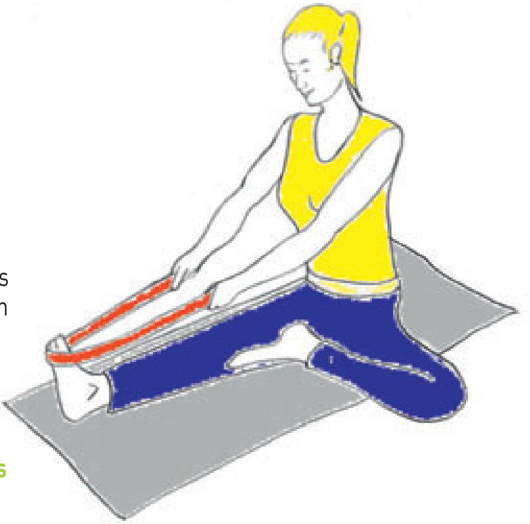

ÉQUILIBRE

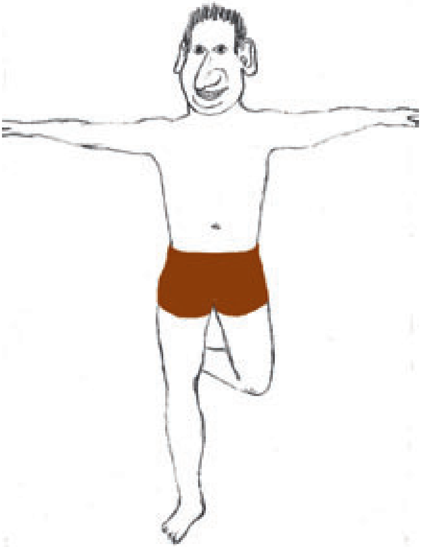

1-Position
Debout

2-Réalisation
En équilibre sur une jambe, genou légèrement fléchi, les bras écartés

VARIANTES
- **Yeux ouverts, puis fermés**
- **Surface dure, souple, coussin**
- **Balancez les bras dans des sens différents**

RÉALISATION DES EXERCICES EN TOUTE SÉRÉNITÉ

- Le travail d'équilibre et de proprioception permet de tonifier et de renforcer le genou.
- Le genou est une articulation intermédiaire, il doit toujours s'adapter aux changements à la fois du sol mais aussi des positions du corps.

Muscles **stabilisateurs**

MOBILITÉ
ÉQUILIBRE
COORDINATION
Réalisez les mouvements avec une grande amplitude
RÉPÉTEZ 10 FOIS CHAQUE EXERCICE

MOBILITÉ ARTICULAIRE

1-Position
Allongé sur le dos

2-Réalisation
Exécutez des mouvements de pédalage dans le vide sans redescendre trop bas, et sans effectuer de flexion complète

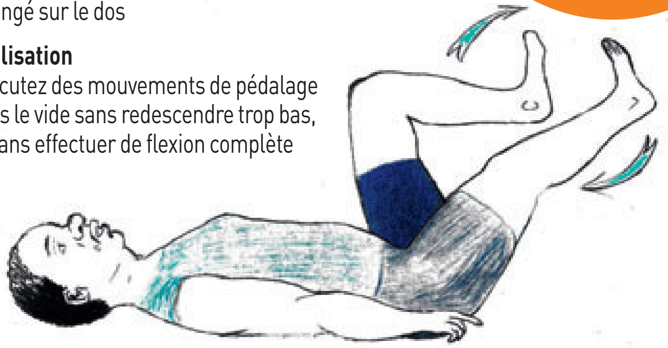

COORDINATION

1-Position
Assise

2-Réalisation
Placez une balle sous le pied et la faire rouler d'avant en arrière

ASTUCE
S'asseoir légèrement en avant de la chaise pour permettre une plus grande amplitude

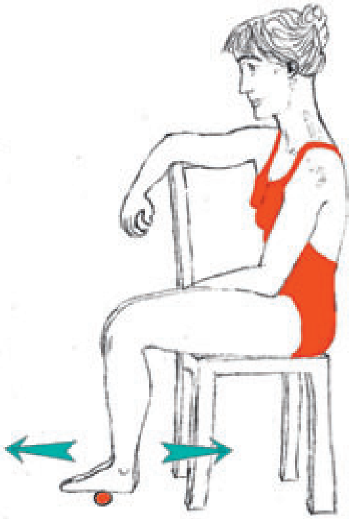

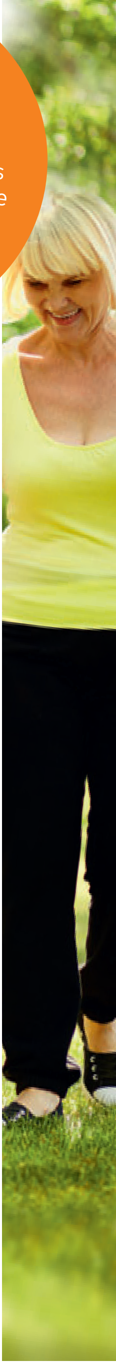

• Un genou tonique se renforce et permet de ménager les autres articulations (chevilles, hanches).
• L'Arthrose réduit la mobilité de vos articulations, d'où la nécessité de l'entretenir.

FICHE 5

Votre choix d'exercices pour l'arthrose du genou

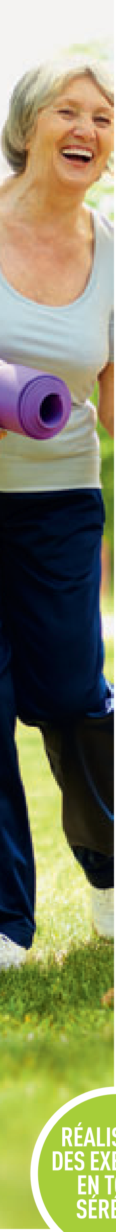

LA MARCHE

AVANTAGES

- Peut être pratiquée dans différentes situations de la vie quotidienne
- Avec des amis ou membres de votre famille

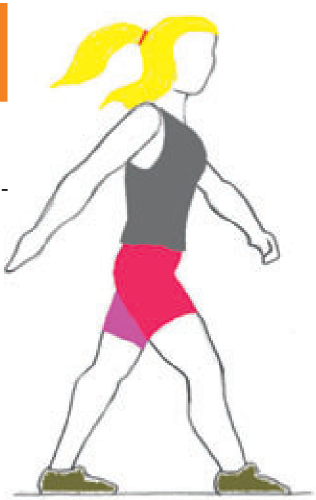

Avec un podomètre, c'est motivant

CONSEIL DOULEURS

- Evitez les terrains trop accidentés afin de protéger vos articulations
- Utilisez des bâtons de marche nordique

LE VÉLO D'APPARTEMENT

AVANTAGES

- On peut faire autre chose en même temps (musique, télévision
- On peut en faire par beau et mauvais temps sans risquer la chute
- Vos genoux ne sont plus soumis à votre poids

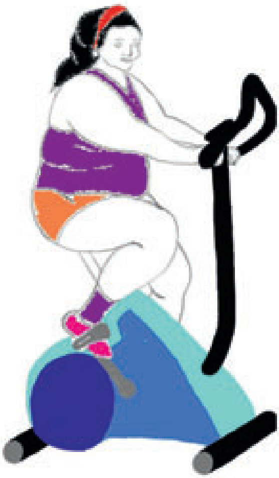

RÉALISATION DES EXERCICES EN TOUTE SÉRÉNITÉ

- N'oubliez pas de commencer par vous échauffer 10 min, augmenter progressivement l'intensité et terminer par un retour au calme de 5 min.

Endurance **cardio-vasculaire**

DURÉE 30 MN
Augmentez progressivement la durée jusqu'à 30 mn

FRÉQUENCE
Au moins 3 à 5 fois par semaine. Restez physiquement actif tous les jours

INTENSITÉ
4 à 6 sur l'échelle de perception. Augmentez graduellement

VÉLO ELLIPTIQUE

AVANTAGES

- Il permet de travailler l'ensemble des muscles du corps, en douceur
- La pratique est similaire à la course à pied
- Il est sans danger pour vos articulations

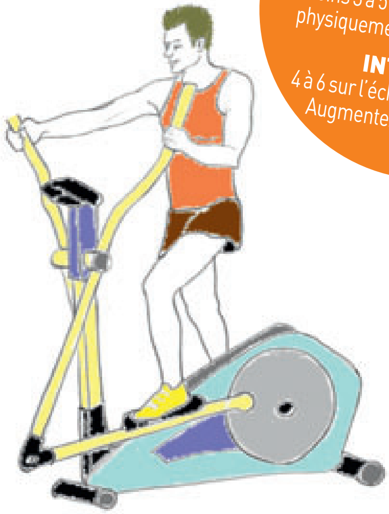

ACTIVITÉS AQUATIQUES

AVANTAGES

- Peut être pratiqué par n'importe quel temps
- Multiples façons de pratiquer (nage, aquagym, aquajogging)
- Vos articulations ne sont plus soumises à votre poids

CONSEIL DOULEURS
Exercices à prioriser en cas de douleurs arthrosiques

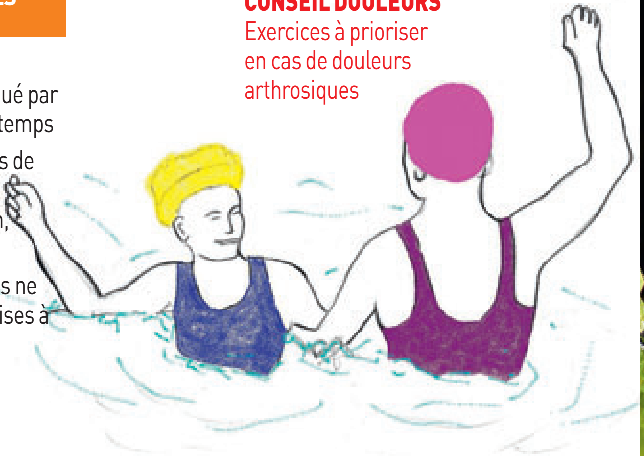

- Votre entraînement peut se faire en continu, en gardant la même intensité tout au long de la période d'exercices. Il peut aussi se faire par intervalles, en alternant des périodes de haute intensité avec des périodes d'intensité plus basse.

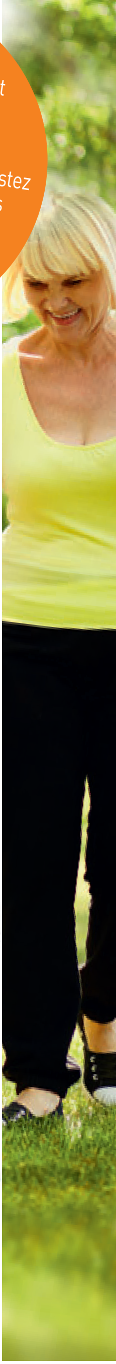

Ce livret a été conçu pour vous aider à bouger au quotidien afin de soulager vos douleurs d'arthrose du genou. Il est issu des données médicales les plus récentes, validées par une équipe pluridisciplinaire.

Ce document a été rédigé par une équipe de spécialistes :

- **Médecine Physique et Réadaptation**
  Médecine du sport
  Pr Emmanuel COUDEYRE, Clermont-Fd
- **Activité Physique Adaptée et Diététique**
  Chloé GAY, Clermont-Fd
- **Médecine Générale**
  Dr Aurore CHABAUD, Clermont-Fd
- **Santé au travail**
  Dr Charlotte LANHERS, Clermont-Fd
- **Rhumatologie**
  Dr Laurent GRANGE
  Président de L'Association Française de Lutte Anti-Rhumatismale

colorteam - 0080C15
